# Perifacial Lymph Node Metastasis After Treatment of Oropharyngeal Primary Malignancy: A Case Report

**DOI:** 10.7759/cureus.31332

**Published:** 2022-11-10

**Authors:** Bailey Hassman, Maranda Thompson, Zafar Sayed

**Affiliations:** 1 Otolaryngology, University of Nebraska Medical Center, Omaha, USA

**Keywords:** tonsillar squamous cell carcinoma, level ib nodes, selective neck dissection, perifacial nodes, oropharyngeal squamous cell carcinoma

## Abstract

With the rate of oropharyngeal cancer on the rise, appropriate surgical management is an increasingly important consideration. Much debate currently exists regarding the necessary extent of neck dissections when performing curative surgery for primary oropharyngeal malignancies. Here, we present the case of a 64-year-old patient with p16+ T1N1M0 squamous cell carcinoma (SCC) of the right tonsil. Approximately four years following transoral robotic surgery oropharyngectomy and ipsilateral level II-IV right selective neck dissection, metastatic SCC was discovered on fine-needle aspiration biopsy of a right perifacial lymph node (level Ib). The patient then underwent a revision right neck dissection at levels Ia and Ib. Adjuvant immunotherapy was recommended following revision neck dissection. Postoperative imaging and flexible laryngoscopy three months after surgery were not concerning for cervical lymphadenopathy or oropharyngeal lesions. Although rare, physicians must maintain a healthy level of suspicion for recurrence to level Ib in oropharyngeal primary malignancies.

## Introduction

The tonsil is the most common site of oropharyngeal cancer, with 23.1% of oropharyngeal malignancies arising at this site [1]. The rate of tonsillar and oropharyngeal malignancies has seen a dramatic increase in the past 40 years due to the increasing prevalence of the human papillomavirus (HPV) [1].

Management of neck metastasis is an important part of the treatment plan for tonsillar squamous cell carcinoma (SCC) given that the presence of neck metastasis is a significant prognostic factor and is associated with a worsening in survival [2]. Current recommendations for the management of the neck in cases of lateralized oropharyngeal carcinoma with clinically negative necks undergoing surgical treatment with curative intent include the completion of selective neck dissection (SND) of ipsilateral levels II to IV, with the inclusion of level Ib up for debate [3-9]. Examining the rate of Ib cervical node positivity and the rate of regional failure in level Ib is important in determining the necessity to include this area in the initial SND.

We present a case report of a patient who was previously treated with transoral robotic surgery (TORS) and SND of levels II-IV with delayed recurrence in a level Ib perifacial lymph node that appeared about four years after the initial surgery.

## Case presentation

The patient is a 64-year-old male former smoker with a history of p16+ pT1N1M0 right palatine tonsil SCC metastatic to the right neck. The patient underwent a TORS tonsillectomy and right limited oropharyngectomy as well as right SND at levels II-IV after diagnosis. The final pathology of the right opharyngectomy specimen revealed a p16+ 1.9 cm primary tumor without perineural invasion or lymphovascular invasion. Margins of the specimen were focally close (<1 mm) but negative for carcinoma. A single lymph node (2.3 cm) out of 23 submitted from the right neck dissection was positive for disease without extranodal extension. After a multidisciplinary tumor board discussion, observation without adjuvant therapy was recommended, and a subsequent postoperative positron emission tomography-computed tomography (PET-CT) was negative for recurrent disease one year after the operation. The patient was also diagnosed with stage III primary kidney cancer and underwent right radical nephrectomy two years following oropharyngectomy and right neck dissection.

**Figure 1:**
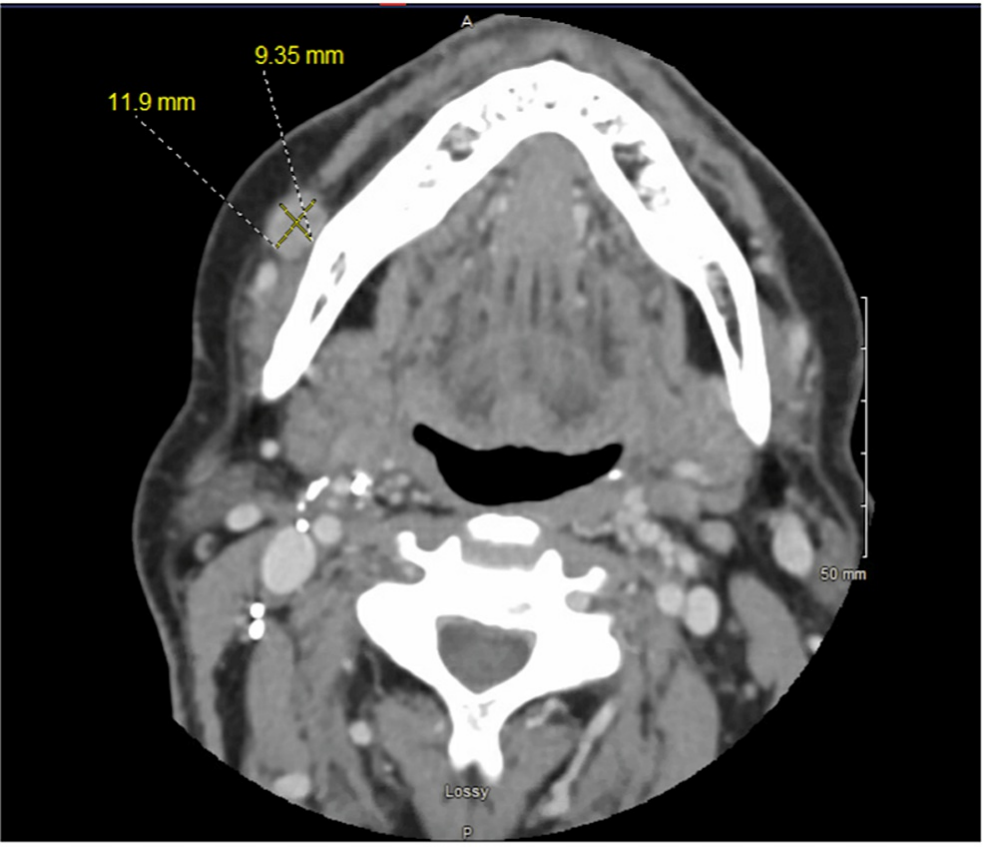
CT with contrast of the neck showing a heterogeneous enhancing 1.2 cm nodule adjacent to the right mandible. CT: computed tomography

**Figure 2:**
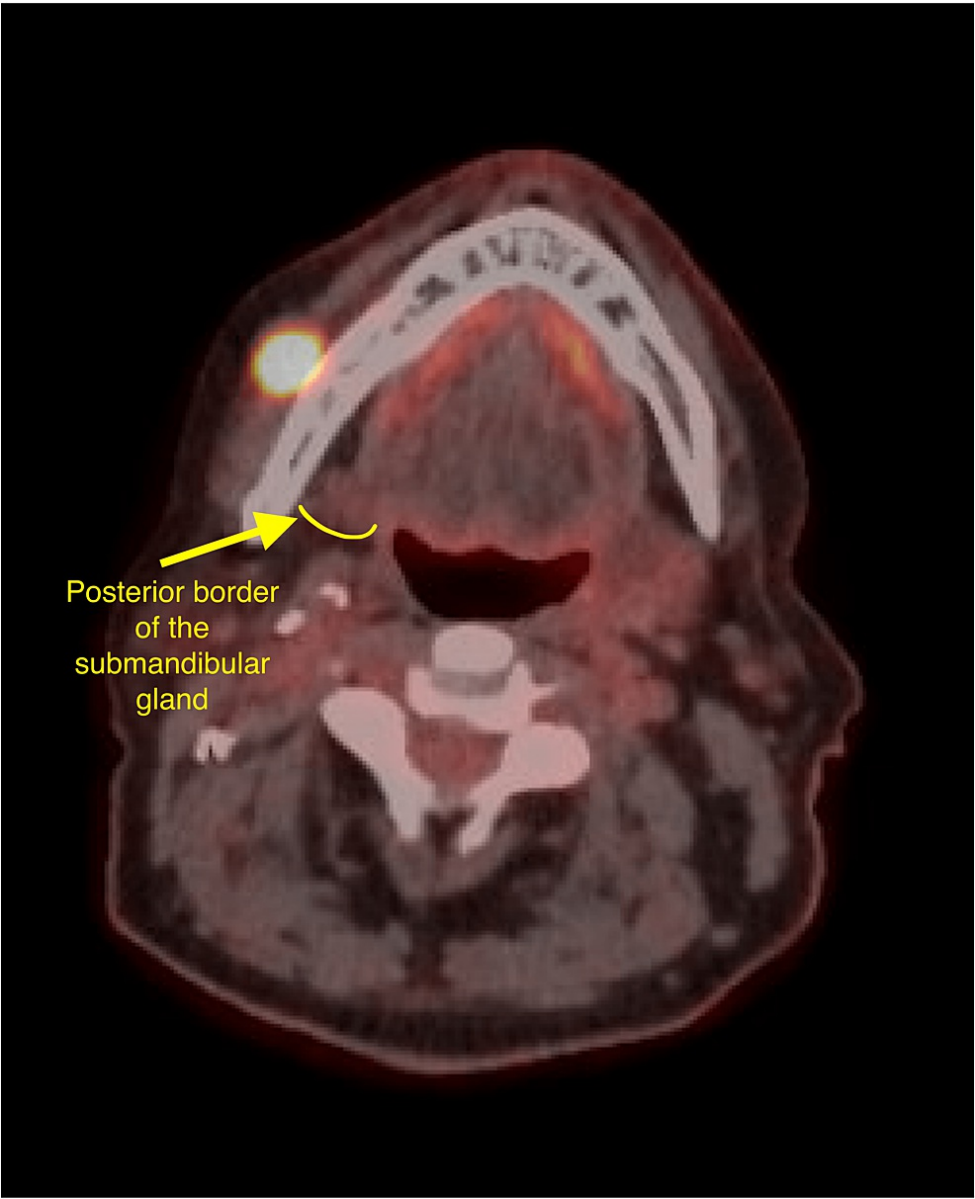
PET-CT axial view three years after initial CT showing a hypermetabolic prominent but non-enlarged level Ib lymph node (SUVmax of 14.0). PET-CT: positron emission tomography-computed tomography; CT: computed tomography; SUVmax: maximum standardized uptake value

**Figure 3:**
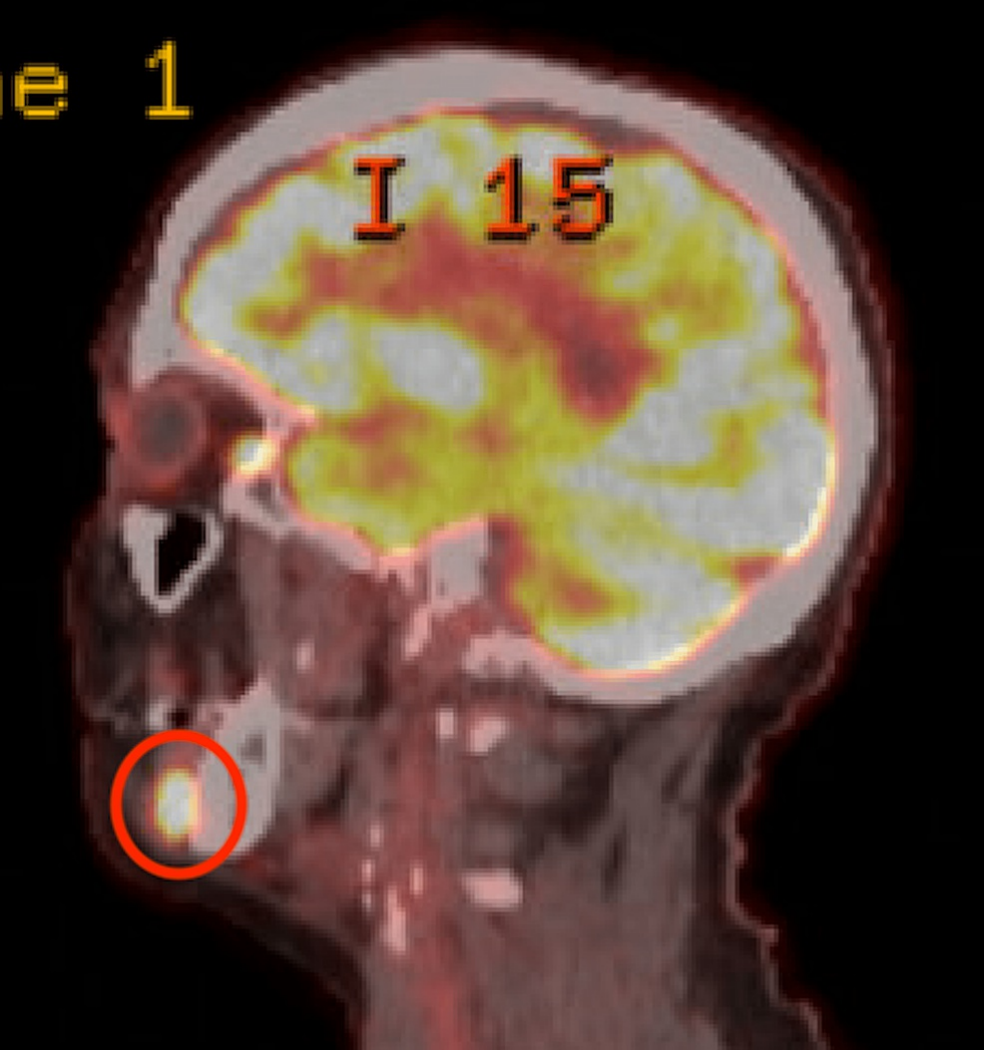
PET-CT sagittal view showing level Ib hypermetabolic lymph node. PET-CT: positron emission tomography-computed tomography

The patient had a long-standing palpable perifacial lymph node overlying the mandibular body that was first noticed following a dental infection. Computed tomography (CT) with contrast of the neck revealed a heterogeneous enhancing 1.2 cm nodule adjacent to the right mandible (Figure 1), prompting ultrasound-guided biopsy. Initial fine-needle aspiration (FNA) biopsy of the perifacial lymph node was performed one year following primary oropharyngeal malignancy diagnosis and surgical treatment, and it demonstrated lymphoid cells negative for malignancy. PET-CT three years after the initial lymph node biopsy showed a hypermetabolic (maximum standardized uptake value = 14.0), prominent but non-enlarged level Ib lymph node, stable in size compared to the prior CT of the neck (Figures 2, 3). Although this perifacial lymph node would not be the expected drainage pathway for an oropharyngeal primary metastasis or renal malignancy, a repeat core needle biopsy of the right perifacial lymph node was performed. Immunohistochemical stains showed the following: p16 (+), p63 (+), p40 (+), CK5/6 (+), CD10 (-), PAX8 (-), and CAIX (+). These findings were consistent with metastatic SCC.

Additionally, the patient completed a course of stereotactic body radiation therapy of his right acetabulum for metastatic renal cell carcinoma in the interval between repeat lymph node biopsy and metastatic lymph node removal.

The patient underwent revision right neck dissection at levels Ia and Ib, including resection of the perifacial lymph node, shortly after the biopsy. Complications included the sacrifice of a distal right marginal mandibular nerve branch due to adherence to the tumor without a clear tissue plane. Pathology of the perifacial lymph node returned as poorly differentiated SCC without extranodal extension. All other cervical nodes were negative for malignancy (0/8). After a second tumor board discussion, adjuvant immunotherapy using pembrolizumab without radiation therapy was recommended given the patient’s history of metastatic renal cell carcinoma.

Postoperative CT of the neck three months after the revision neck dissection revealed no additional cervical lymphadenopathy, and flexible laryngoscopy was without concerning oropharyngeal findings.

## Discussion

While alcohol and tobacco are well-known risk factors for oropharyngeal cancer, HPV can now be identified in more than 70% of new cases of oropharyngeal cancer in the United States [10]. Oral HPV16 is responsible for the overwhelming majority of HPV-related oropharyngeal cancers, and over 90% of these infections are sexually transmitted [11]. Additionally, HPV16 infections are much more prevalent in men (as much as six times) than in women, and they occur more often in individuals of higher socioeconomic status without histories of smoking or alcohol use [12,13]. With HPV-related oropharyngeal cancer now the most common HPV-related malignancy in the United States, prevention and treatment are major national healthcare issues [14]. Several studies have shown that HPV vaccination can be very effective in the prevention of oral HPV infections, prompting the Food and Drug Administration to recently expand the indication of HPV vaccines to include oropharyngeal cancer prevention [11,15]. Based on the latest HPV vaccination coverage estimates in 2017, 51% of adolescents have not completed the HPV vaccination series, with rural adolescents receiving fewer HPV vaccines than their urban counterparts [16]. Without a reliable screening test or any premalignant lesions for early detection, expanding the use of HPV vaccination is crucial in reducing the number of patients affected by oropharyngeal cancer.

Along with the prevention of oropharyngeal cancer, effective treatment is critical in reducing patient morbidity and mortality. Current National Comprehensive Cancer Network (NCCN) Guidelines include SND of level II-IV when performing surgical treatment with curative intent for lateralized oropharyngeal carcinoma, which is the initial treatment this patient received [9]. The inclusion of level Ib in this dissection has been heavily debated [3-9]. According to the widely used Robbin’s classification, level Ib corresponds to the submandibular lymph nodes within the boundaries of the anterior belly of the digastric muscle, the body of the mandible superiorly, and the posterior border of the submandibular gland [17,18]. Within level Ib, five groups of submandibular nodes have been described based on their relationship to the anterior facial vein and the submandibular gland: preglandular, retroglandular, intraglandular, prevascular, and retrovascular [19]. Perifacial nodes refer to prevascular and retrovascular lymph nodes surrounding the anterior facial vein; the specific node that contained metastatic SCC in this patient was classified as a perifacial node [18]. The decision to exclude level Ib from the neck dissection when treating oropharyngeal carcinoma is based on the findings that level I, including the submental and submandibular regions, has not been classically recognized as a drainage pathway for tumors of the oropharynx thereby it is less likely to contain metastatic disease [7,20]. Furthermore, the addition of level I, specifically level Ib, to SND brings a higher risk of marginal mandibular nerve injury, as experienced by our patient, and oropharyngocutaneous fistula with the removal of the submandibular gland [5].

To determine whether additional level I dissection needs to be performed, several studies have examined the rate of metastasis to level I from oropharyngeal primary sites. Lim et al. found that only 10% out of 68 patients who underwent therapeutic ipsilateral neck dissection had positive lymph nodes at level I [21]. These findings were echoed in a study by Da Mosto et al. who observed no nodal metastases in level I in elective or therapeutic neck dissection out of 58 patients with previously untreated tonsillar SCC [22]. Another study by Lim et al. found that the incidence rate of metastasis to perifacial lymph nodes in patients with primary oropharyngeal carcinoma and clinically node-positive neck was 8% and 6%, respectively, without clinically positive level I nodes [23]. These findings show that metastasis to level I lymph nodes is very rare in primary oropharyngeal cancer, supporting current treatment recommendations and confirming that this patient received the appropriate initial surgical management.

Studies have also examined the rate of recurrence to level I lymph nodes after primary lesion removal and SND in patients with oropharyngeal SCC, which reflects the scenario presented in this case. In a study of 58 patients with tonsillar SCC treated with neck dissections and with/without adjuvant radiation treatment, only one patient who had undergone level I-V dissection for T2N0 tonsillar SCC developed recurrent carcinoma in ipsilateral level I [22]. Lim et al. studied 104 patients with oropharyngeal cancer who were treated with neck dissections and primary tumor resections and observed two cases of recurrence in level I after treatment [21]. Cannon et al. found that out of 88 patients with p16+ oropharyngeal SCC who were treated with TORS and neck dissection at level II-IV, no regional failures occurred in level Ib nodes [5]. These findings show that the rate of recurrence in level I lymph nodes after treatment of primary oropharyngeal malignancies is very low, highlighting the rarity of this case. Although infrequent, clinicians should maintain a healthy level of suspicion for metastasis to level I lymph nodes in oropharyngeal malignancies.

## Conclusions

This case report of an unusual recurrence to a level Ib perifacial lymph node from a tonsillar primary SCC highlights a unique pattern of metastasis from an oropharyngeal primary site. Several studies have determined that the rate of metastasis and recurrence after treatment to level I lymph nodes from primary oropharyngeal SCC is very rare; therefore, it is likely not necessary to include level Ib in the initial SND when performing curative surgery for oropharyngeal malignancy. Although infrequent, this case shows that physicians must maintain an appropriate level of suspicion for metastasis from oropharyngeal primary malignancies to level Ib lymph nodes.
